# Bpur, the Lyme Disease Spirochete's PUR Domain Protein: IDENTIFICATION AS A TRANSCRIPTIONAL MODULATOR AND CHARACTERIZATION OF NUCLEIC ACID INTERACTIONS*

**DOI:** 10.1074/jbc.M113.491357

**Published:** 2013-07-11

**Authors:** Brandon L. Jutras, Alicia M. Chenail, Dustin W. Carroll, M. Clarke Miller, Haining Zhu, Amy Bowman, Brian Stevenson

**Affiliations:** From the ‡ Department of Microbiology, Immunology, and Molecular Genetics and; the § Graduate Center for Toxicology, University of Kentucky College of Medicine, Lexington, Kentucky 40536,; the ¶ James Graham Brown Cancer Center, University of Louisville, Louisville, Kentucky 40202, and; the ‖ Department of Molecular and Cellular Biochemistry, University of Kentucky College of Medicine, Lexington, Kentucky 40536

**Keywords:** Bacteria, DNA-binding Protein, Gene Regulation, RNA-binding Protein, Transcription Regulation, Borrelia burgdorferi, PUR Domain, erp

## Abstract

The PUR domain is a nucleic acid-binding motif found in critical regulatory proteins of higher eukaryotes and in certain species of bacteria. During investigations into mechanisms by which the Lyme disease spirochete controls synthesis of its Erp surface proteins, it was discovered that the borrelial PUR domain protein, Bpur, binds with high affinity to double-stranded DNA adjacent to the *erp* transcriptional promoter. Bpur was found to enhance the effects of the *erp* repressor protein, BpaB. Bpur also bound single-stranded DNA and RNA, with relative affinities RNA > double-stranded DNA > single-stranded DNA. Rational site-directed mutagenesis of Bpur identified amino acid residues and domains critical for interactions with nucleic acids, and it revealed that the PUR domain has a distinct mechanism of interaction with each type of nucleic acid ligand. These data shed light on both gene regulation in the Lyme spirochete and functional mechanisms of the widely distributed PUR domain.

## Teaser

**Background:** *Borrelia burgdorferi* controls protein production during its infectious cycle.

**Results:** Bpur was identified and shown to enhance effects of the *erp* transcriptional repressor, BpaB. Details of Bpur-nucleic acid interactions were obtained.

**Conclusion:** Bpur participates in regulation of protein expression.

**Significance:** A new bacterial regulatory factor was identified and insight garnered on mechanisms by which Bpur interacts with nucleic acids.

## Introduction

The Lyme disease bacterium, *Borrelia burgdorferi*, persists in nature through a two-host cycle involving vertebrates and ticks (1). This complex infectious cycle requires that the bacteria not only efficiently colonize two very different types of hosts but must also move between them. Through largely unknown mechanisms, *B. burgdorferi* senses its environment during the cycle and, in response, controls production of many different surface proteins. Deciphering *B. burgdorferi's* regulatory networks is casting light on the infectious mechanisms of the spirochete and identifying new targets for antibacterial therapies. In addition, the Lyme disease spirochete is a genetically tractable model vector-borne pathogen and, as demonstrated herein, can also be an excellent model for studies of ubiquitous regulatory factors.

**FIGURE 1.**
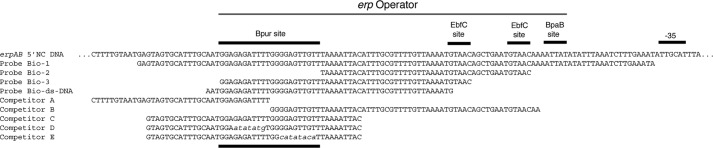
**Sequences of nucleic acids used in these studies.** For dsDNAs, only the forward strand sequences are shown. The *top line* shows the 5′-noncoding sequence of the *B. burgdorferi* strain B31 *erpAB* operon from the operator through the −35 sequence of the promoter. The region bound at high affinity by Bpur is illustrated, as are the previously defined high affinity binding sites of EbfC and BpaB (17–19). Below that are shown the sequences of EMSA probes and competitors. Those with names that include *Bio* were 5′-end-labeled with biotin. The sequences of biotin-labeled ssDNA and RNA probes were based on that of probe Bio-dsDNA. Mutated bases in competitors C and D are indicated by *lowercase italics*.

*B. burgdorferi* produces Erp^2^ outer surface lipoproteins throughout mammalian infection, but it represses their synthesis during tick colonization (2–4). Known functions of Erp proteins include binding of host plasmin(ogen), laminin, and the complement regulator factor H (5–13). A highly conserved DNA region immediately 5′ of all *erp* promoters, the *erp* operator, is required for regulation of *erp* transcription (Fig. 1) (14, 15). Previous DNA-affinity chromatography identified two borrelial cytoplasmic proteins that bind with high affinities to *erp* operator DNA as follows: BpaB (borrelial plasmid ParB analog) and EbfC (*e**rp*-binding factor, chromosomal) (16–21). Binding of BpaB to the *erp* operator represses transcription, whereas EbfC competes with BpaB for DNA binding and thereby acts as an antirepressor (17, 20). Those earlier DNA affinity assays indicated that an additional borrelial protein bound to the *erp* operator (16). We now present identification of the third *erp* operator-binding protein, Bpur (borrelial PUR domain protein), evaluation of its effects on *erp* expression, and characterization of mechanisms by which Bpur interacts with nucleic acids.

**FIGURE 2.**
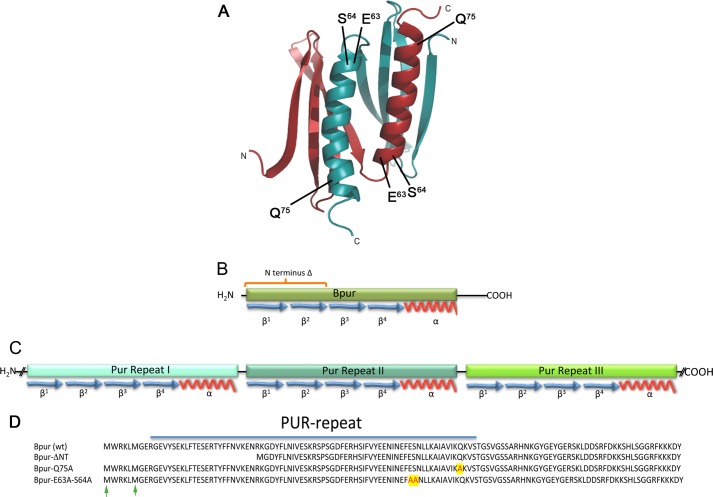
**Structural characteristics of *B. burgdorferi* Bpur and human Pur-α.** *A,* three-dimensional structure of Bpur has been solved, and its PUR domain is very similar to those of eukaryotic proteins (22). The diagram illustrates amino acid residues and structural regions of Bpur demonstrated by the current studies to be involved with ligand binding. Each monomer folds into a PUR repeat of four anti-parallel β-strands and a single α-helix, which dimerize to form the PUR domain (22, 23). In the diagram, one subunit is colored *blue*, and the other is *red*. Residue glutamine 75 was mutated to alanine to produce mutant protein Bpur-Q75A. Residues glutamate 63 and serine 64 were both mutated to alanine to produce protein Bpur-E63A/S64A. *B,* linear diagram of one Bpur monomer, indicating secondary structures. Bpur contains only 122 amino acids, which form a single PUR-repeat, and two Bpur molecules homodimerize to form a functional PUR domain (22). *C,* linear diagram of the human Pur-α protein, illustrating the protein's three PUR domains and connecting regions. Based on the solved structure of the *D. melanogaster* homolog, it is predicted that PUR repeats 1 and 2 form an intramolecular PUR domain, whereas PUR repeat 3 is thought to be involved with multimerization (23). Those features pose significant difficulties to the use of eukaryotic homologs for structure-function studies of PUR domain proteins, and because each PUR repeat is unique, individual mutations change only one-half of the PUR domain, and the physical linkage of the PUR-repeats means that deletions can disrupt folding of the entire protein. *D,* amino acid sequences of wild-type Bpur and the Bpur mutants that exhibited significantly impaired ligand binding activities. Altered residues are highlighted by *red* on *yellow*. Alternate initial methionine residues are indicated by *green arrows*.

Serendipitously, while our studies of Bpur function were underway, the three-dimensional structure of Bpur was solved (Fig. 2*A*) (22). The rationale for determining Bpur's structure was that it contains a PUR domain, a motif composed of two interwoven “PUR repeat” sequences, each of which consists of four antiparallel β-strands and a single α-helix (Fig. 2) (22, 23). PUR domain proteins are so-named because that motif binds purine-rich sequences in single-stranded (ss) and double-stranded (ds) DNA and in RNA. However, very little is known about the mechanisms by which the PUR domain interacts with its nucleic acid ligands. Proteins with PUR domains are found throughout nature, from single-cell bacteria to complex eukaryotes such as humans. They are known to be key regulatory factors of vertebrates, insects, trematodes, and plants (24–52). The human PUR proteins play roles in numerous diseases, including cancers, fragile X tremor/ataxia syndrome, and Alzheimer disease (24–29), and in replication of viruses such as HIV-1 and JCV (30–32). Mice deficient in the PUR-α protein exhibit severe nervous system deficits and die within 2 weeks after birth (25, 33–35). The earlier structural study of Bpur found that this protein has a moderate affinity for an ssDNA sequence that can also be bound by the human PUR-α, although the ability of the borrelial protein to bind either dsDNA or RNA was not examined (22). Bpur contains only 122 amino acids and folds into a single PUR repeat with short, unstructured amino and carboxyl termini (22). Two Bpur polypeptides dimerize to form the functional protein. This is significantly less complex than eukaryotic PUR domain proteins, which consist of three PUR repeats, each with a different amino acid sequence, forming inter- and intra-molecular PUR domains, plus containing substantial lengths of linking sequences (Fig. 2) (22, 23). Just as Bpur served as a model to help determine the structures of the more complex eukaryotic PUR domain proteins (22, 23), we took advantage of Bpur's small size, absence of superfluous linking polypeptides, and the identical nature of both halves of the PUR domain to investigate the mechanisms by which a PUR domain interacts with nucleic acids. Thus, insight was obtained not only on how Bpur influences *erp* expression but also on the mechanics underlying interactions between the PUR domain and its ligands.

## EXPERIMENTAL PROCEDURES

### 

#### 

##### Identification of Bpur

Bpur was identified using DNA affinity chromatography, following previously described procedures (16, 53). Briefly, an infectious clone of *B. burgdorferi*, strain B31-MI-16 (2), was grown to mid-exponential phase (∼5 × 10^7^ bacteria/ml) at 34 °C in Barbour-Stoenner-Kelly II medium (54). Bacteria were harvested by centrifugation, washed gently with phosphate-buffered saline (PBS), pelleted, and then subjected to two −80 °C/ice freeze and thaw cycles. The partly lysed bacteria were gently resuspended in a 20:1 mix of BS/THES buffer (53) and B-PER II protein extraction reagent (Thermo-Fisher) and then incubated with rocking at room temperature for 30 min. Lysates were cleared by centrifugation at 17,000 × *g* for 30 min at 4 °C. Aliquots of cytoplasmic extracts were stored at −80 °C.

Biotin-labeled 5′-noncoding DNA from the *B. burgdorferi* B31-MI-16 cp32-1 *erpAB* operon was PCR-amplified as described previously (16). The amplicon was purified by agarose gel electrophoresis, re-amplified, and then concentrated in nuclease-free water at 250 ng/μl. The biotinylated bait DNA was bound to streptavidin Dynal beads (Invitrogen) and then washed with BS/THES buffer. Borrelial cytoplasmic extracts were incubated with the DNA beads and washed extensively with BS/THES, taking advantage of the Dynal beads magnetic properties when changing buffers. Bound proteins were eluted by increasing NaCl concentrations to 200, 300, 500, 750, and 1000 mm. Fractions of the eluates were analyzed by SDS-PAGE, and proteins were stained with SYPRO-Ruby (Molecular Probes, Eugene, OR). Protein bands were excised and analyzed by matrix-assisted laser desorption ionization/time of flight at the University of Louisville Clinical Proteomics Center (Louisville, KY). Results were compared with the *B. burgdorferi* strain B31-MI sequence (55, 56) using Mascot (Matrix Science, Boston). Significance parameters were fixed at *p* < 0.05, corresponding to Ion score of 81.

##### Recombinant Proteins

Bpur is encoded by the previously undescribed open reading frame BB0047 of *B. burgdorferi* type strain B31 (55). The *bpur* open reading frame was PCR-amplified from *B. burgdorferi* B31 genomic DNA and cloned in pET101 (Invitrogen) to produce pBLJ210. Subsequent mutant *Bpur* genes, encoding truncated proteins, were produced by overlap extension PCR mutagenesis of pBLJ210 (57). All mutant proteins were prepared, purified, and otherwise treated in an identical manner. Recombinant proteins produced and analyzed during the course of this work are described in Fig. 2 and Table 1.

Recombinant proteins were produced using *Escherichia coli* strain Rosetta-gami 2 (Novagen). Induced bacteria were harvested by centrifugation, washed with PBS, and lysed by sonication, and cellular debris was removed by centrifugation. Recombinant proteins were purified from cleared lysates using MagneHis nickel particles (Promega, Madison, WI). For all electrophoretic mobility shift assays (EMSA), purified proteins were dialyzed with DNA-binding buffer (100 nm dithiothreitol (DTT), 50 mm Tris (pH 7.5), 25 mm KCl, 1 mm MgCl_2_, 10% glycerol (v/v), 0.01% Tween 20, 0.1% phenylmethanesulfonyl fluoride). For use in limited trypsin protease protection assays, rBpur was dialyzed against a buffer containing 20 mm Tris-HCl (pH 7.5), 100 nm DTT, 0.01% Tween 20, 1% glycerol. In all cases, protein concentrations were determined by Bradford assay (Bio-Rad). Purities were determined by SDS-PAGE and stained with Coomassie Brilliant Blue. Aliquots were stored at −80 °C.

##### Antiserum Production

Antiserum was raised against Bpur in New Zealand White rabbits and affinity-purified by a commercial vendor using their standard protocols (NeoBioscience). For use in control studies, samples of blood were collected from the rabbits before immunization, then pooled, and processed into serum (“preimmune serum”).

Antiserum directed against full-length recombinant SsbP was produced using BALB/c mice. Preimmune serum was collected before initiation of the injection protocol. Mice were injected subcutaneously with 10 μg of recombinant SsbP in 80 μl of 60% AlOH (mass/volume), followed by two additional injections 2 weeks apart. One week after the final boost, mice were euthanized, and their blood was pooled and processed into serum.

##### Electrophoretic Mobility Shift Assays (EMSA)

Labeled nucleic acid probes and unlabeled competitors are described in Fig. 1. dsDNA probes and competitors were generated either by PCR or by annealing synthetic oligonucleotides, as described previously (19, 20). For dsDNA probes, one oligonucleotide primer was 5′-end-labeled with biotin (Integrated DNA Technologies (IDT), Coralville, IA). 5′-End-labeled ssDNA and RNA probes were synthesized chemically by IDT.

For RNA binding assays, all equipment was treated with diethyl pyrocarbonate prior to use, and RiboGuard (Epicenter, Madison, WI) was added to each reaction to a final concentration of 0.01 μg/ml. For assays of RNA and ssDNA binding, the nucleic acid probes were incubated at 56 °C prior to protein-substrate reactions, to destabilize secondary structures.

EMSAs were performed essentially as described previously (17, 18). Protein-nucleic acid combinations were subjected to electrophoresis using pre-cast 6 or 10% nondenaturing polyacrylamide gels (Invitrogen). Following transfer to Biodyne nylon membranes (Thermo Pierce) and UV cross-linking (Stratalinker 1800, Stratagene, San Diego), biotin-labeled DNAs were visualized using nucleic acid detection kits (Thermo Pierce) and autoradiography. Band intensities were quantified using ImageJ (rsbweb.nih.gov).

Dissociation constants (*K_D_*) were determined by analyses of EMSA gel images, as described previously (18, 58–60). Exposed films were scanned and analyzed using ImageJ (61). Graphical representations of Bpur-substrate interactions were performed using GraphPad Prizm and calculated as described previously (18). The ratio of free to bound nucleic acid was calculated for each reaction and a mean was determined and adjusted depending upon substrate concentrations to allow for independent comparisons.

In the case of cold competitor EMSAs, unlabeled dsDNAs were generated by PCR or by annealing synthetic oligonucleotides (19). Unlabeled DNAs were added to ×100 molar excess over those of labeled probe DNAs. Equal concentrations of labeled probes were added to all simultaneous EMSA reactions.

Anti-SsbP supershift EMSAs were performed as above, with an additional preincubation for 8 min with nuclease-free water, preimmune serum, or SsbP-directed antiserum. DTT was omitted from all buffers for supershift reactions.

##### In Vitro Coupled Transcription/Translation

A linear DNA fragment consisting of 471 bp of *erpAB* 5′-noncoding DNA fused to *gfp* was used to measure *erp* promoter activity, as described previously (20). Bovine serum albumin (BSA) was used for some control experiments. Reactions used the cell-free *E. coli* S30 Extract Transcription/Translation System for Linear Templates (Promega). Each 75-μl reaction contained 105 nm DNA template, 160 nm of each protein (alone or together, as well as no added protein), 4 mm NaCl, 4 mm Tris-HCl, 80 mm NaHPO_4_, 0.75 nm DTT, in the following volumes of kit reagents: 30 μl of S30 Premix, 22.5 μl of *E. coli* S30 extract, and 7.5 μl of 1 mm amino acid mixture. To ensure that experimental readouts were due to *gfp* transcription, rifampin was added to control reactions at a final concentration of 40 mg/ml. Additional control experiments replaced the DNA with 6 μl of nuclease-free water. Reactions were lightly mixed and incubated at 37 °C for 80 min. Reactions were stopped by incubation on ice for 15 min, and proteins were precipitated with acetone and then resuspended in 85 μl of PBS.

For ELISA, 60 μl of resuspended products were added to 380 μl of ELISA coating buffer (50 mm Na_2_CO_3_, 500 mm NaHCO_3_ (pH 9.2)). GFP product was measured using MACS molecular anti-GFP:horseradish peroxidase conjugate (Miltenyi Biotec, Auburn, CA) and Turbo TMB ELISA (Thermo-Fisher, Pittsburgh, PA). Reactions were stopped with 2 n H_2_SO_4_, and absorbance at 450 nm was measured with a VersaMax tunable microplate reader. Each experiment was performed with five simultaneous, identical reactions, and all experiments were replicated at least three times.

##### In Vivo Induction of Bpur from an Inducible Plasmid Construct

The previously described pSZW53-4 replicates autonomously in both *B. burgdorferi* and *E. coli*, and it contains both a constitutively expressed *tetR* gene and a TetR-repressible promoter, P*ost* (20, 62). The *bpur* gene was cloned into that vector such that its transcription was dependent upon the inducible P*ost* promoter. The resultant chimeric plasmid was introduced into *B. burgdorferi* strain B31-e2 by electroporation. Transcription from the P*ost* promoter was induced by addition of anhydrotetracycline, at a final concentration of 0.5 mg/ml, to early exponential phase cultures (∼10^5^ bacteria/ml). After cultivation to final densities of ∼10^7^ bacteria/ml, bacteria were harvested by centrifugation and lysed, and proteins were separated by SDS-PAGE. Total proteins were detected by Coomassie Brilliant Blue staining. Individual proteins were identified by immunoblot using monospecific antibodies (63) and analyzed densitometrically.

##### Changes of Culture Conditions

All studies utilized *B. burgdorferi* strain B31-MI-16. Temperature-shift experiments from 23 to 34 °C were performed as described previously (64, 65). Briefly, *B. burgdorferi* was first cultured to late exponential phase (∼10^8^ bacteria/ml) in BSK-II at either 23 or 34 °C. An aliquot of such a culture was diluted 1:100 into fresh BSK-II and then incubated at 23 °C. Upon that culture attaining late exponential phase, an aliquot was diluted 1:100 into fresh medium and then incubated at 34 °C. Late exponential phase cultures of the constant 23 °C and the 23 to 34 °C-shifted bacteria were harvested for analyses.

The effects of culture medium composition were assessed using essentially the same technique (66). Bacteria were grown to late exponential phase at 34 °C in an incomplete medium. An aliquot of that culture was then diluted 1:100 into fresh, complete BSK-II and then incubated at 34 °C. Both cultures were harvested at late exponential phase. Two incomplete culture media were used. Complete BSK-II contains 6% rabbit serum, which provides the bacteria with lipids, and BSK-II containing only 1.2% rabbit serum reduces growth rate by approximately one-third (66). A similarly reduced growth rate is obtained with BSK-II that has been diluted to one-quarter strength and includes 6% rabbit serum (66).

Total cellular proteins were separated by SDS-PAGE. Specific proteins were detected by immunoblot analyses using either the above-described Bpur-directed antiserum or a murine monoclonal antibody specific for ErpA (63). The constitutively expressed FlaB protein served as a reference (65, 67).

##### Bpur Modeling

Modeling of Bpur and *Drosophila* Pur-α structures were performed using RaptorX and Jmol. Structural information on *B. burgdorferi* Bpur and *Drosophila melanogaster* Pur-α were obtained from the Research Collaboratory for Structural Bioinformatics Protein Data Bank files 3N8B and 3K44, respectively.

##### Bpur/SsbP Co-immunoprecipitation

To determine whether Bpur and SsbP physically interact with each other, co-immunoprecipitation assays were performed. Briefly, equal concentrations of purified SsbP and Bpur (1 μm each) were incubated together at room temperature and then applied to Bpur-directed antiserum bound to protein A-conjugated resin beads (Invitrogen). Control reactions included incubation of Bpur and SsbP with protein A-conjugated beads or with goat anti-mouse IgG (Santa Cruz Biotechnology) bound to protein A-conjugated beads. Immunoprecipitation was carried out as described previously (21). Antigens were eluted from experimental and control beads. Each eluate, plus additional controls of purified Bpur and SsbP, was separated by SDS-PAGE and transferred to a nitrocellulose membrane. Membranes were probed with antiserum specific for either Bpur or SsbP. Antibodies were detected via HRP-conjugated secondary antibodies and detected using chemiluminescence (Thermo Scientific).

##### Limited Trypsin Proteolysis

Recombinant Bpur, at a final concentration of 50 nm, was incubated with a saturating excess of a nucleic acid ligand (2 μm either dsDNA, ssDNA, or RNA), in a total volume of 85 μl of buffer that consisted of 20 mm Tris-HCl (pH 7.0), 100 nm DTT, 0.001% Tween 20 (v/v), and 5% glycerol (v/v). Control reactions included the same concentration of Bpur without a nucleic acid ligand. All constituents were allowed to come to equilibrium at room temperature for 8 min. Trypsin was then added to a final concentration of 1 nm. A 15-μl aliquot was removed after 5, 10, 20, and 30 min. Reactions were stopped by immediately snap freezing the aliquots in liquid nitrogen.

The resulting polypeptides were diluted by dissolving 10 μl of sample in 90 μl of 0.1% formic acid (v/v). Samples were then filtered through 0.45-μm low protein-binding Durapore PVDF syringe-driven filters (Millipore, Billerica, MA) by centrifugation at 1500 rpm for 4 min. Aliquots (5 μl) of each sample were injected for nano-LC-MS/MS analysis. LC-MS/MS data were acquired on an LTQ Velos Orbitrap mass spectrometer (Thermo Fisher Scientific, Waltham, MA) coupled with a nano-LC Ultra/cHiPLC-nanoflex HPLC system (Eksigent, Dublin, CA) through a nano-electrospray ionization source. The tryptic polypeptide solutions were separated via automated injection, trap column desalination, and reverse phase chromatography using a C18 column (75 μm inner diameter × 150 mm). A 50-min HPLC gradient was run at a flow rate of 300 nl/min as follows: 0–24 min, 3–40% B; 24–27 min, 40–95% B; 27–36 min held at 95% B; 36.1–50 min, held at 3% B (mobile phase A = 100% H_2_O, 0.1% formic acid; mobile phase B = 100% acetonitrile, 0.1% formic acid). Two blank (50% A and 50% B) injections of 5 μl were run between each sample injection. Eluted polypeptides were characterized using data-dependent acquisition; polypeptide mass spectra ranging from *m*/*z* 300 Da to *m*/*z* 1800 Da were obtained via Orbitrap analysis, with a resolution of 60,000.

The seven most abundant polypeptides identified in the Fourier transform-MS survey scan were then subjected to collision-induced dissociation and MS/MS analysis in the LTQ ion trap. The data were submitted to a local MASCOT server, which used the sample MS/MS spectra to search a custom database containing only the amino acid sequence of Bpur, for MS/MS protein identification using the Proteome Discoverer 1.3 software (Thermo Fisher Scientific, Waltham, MA). The mass error tolerance was 20 ppm for polypeptide MS analysis and 0.8 Da for MS/MS analysis.

Tryptic polypeptides identified by MASCOT search via Proteome Discoverer 1.3 were mapped against the amino acid sequence of Bpur for each time point and substrate group. Ion scores pertaining to individual polypeptide concentrations and the confidence of polypeptide identification through the analysis of MS/MS spectra were used to list the identified polypeptides within the map. Those polypeptides with the lowest ion scores were deemed to be present at the lowest concentrations. Cleavage sites were identified through the evaluation of the presence/absence of individual polypeptides or the increase/decrease of individual polypeptide ion scores between various substrate groups and time points. Extracted ion chromatograms for polypeptides surrounding a given cleavage site of interest were evaluated for signal intensity as well as peak area. These values were used to assess differential polypeptide cleavage between substrate groups. Differential polypeptide cleavage between substrate groups was then used to evaluate the relative protection or exposure of a cleavage site due to substrate binding.

All cleavage events were normalized against reactions lacking nucleic acid substrate at the same time point. Values were log_2_-transformed, and decreases in polypeptide presence were divided by −0.1 to assign negative values. Graphical representations of the data were first evaluated using R64 and adjusted using Microsoft PowerPoint with Macros. Sites with no difference or noncleavage sites were labeled black by default and assigned a value of 1.

## RESULTS

### 

#### 

##### Identification of Bpur and a High Affinity Nucleic Acid-binding Sequence

**FIGURE 3.**
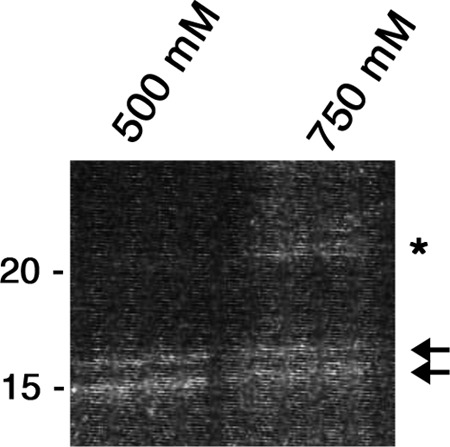
**DNA-affinity chromatography resulted in purification of Bpur.** Biotinylated DNA was affixed to magnetic beads and incubated with crude cytoplasmic extract from *B. burgdorferi* that had been cultured to mid-exponential phase. Following extensive washing, proteins were eluted from the DNA with increasing concentrations of NaCl, then separated by SDS-PAGE, and stained with Sypro Ruby. Proteins that eluted at 500 and 750 mm NaCl are shown. Both of the ∼17-kDa proteins marked with *arrowheads* were identified by mass spectrometry as being Bpur. Additional studies determined that translation of Bpur can initiate from two AUG start codons, located 15 bases apart on the same mRNA. The *asterisk* indicates the previously characterized BpaB protein, which also binds to this bait DNA (17).

Our earlier studies found that *B. burgdorferi* produces at least three cytoplasmic proteins that bind with high affinities to DNA adjacent to the *erp* transcriptional promoter (16). Two of those proteins were subsequently identified as the BpaB repressor and the nucleoid-associated protein EbfC, which also functions as the *erp* antirepressor (16–18, 20, 21). Repeats of those experiments using higher concentration polyacrylamide gels resolved the previously unidentified PAGE band into two protein bands of ∼17 kDa (Fig. 3). Mass spectrometric analyses identified both proteins to be the product of locus BB0047 of *B. burgdorferi* type strain B31 (55). With significance parameters fixed at *p* < 0.05, the slowest migrating species produced an ion score of 122, whereas the faster migrating species produced a score of 238. Repetition of the DNA affinity chromatography and mass spectrometry yielded essentially the same results. The gene was given the name *bpur* (borrelial *PUR*) in consequence of the encoded protein's PUR domain. All sequenced isolates of Lyme disease-associated *Borrelia* spp. maintain an identical *bpur* gene (55, 68–74).

The *bpur* ribosome-binding site is followed by two in-frame AUG start codons, which are separated by 15 bases. Two plasmids were constructed in which *bpur* was placed under an inducible promoter, one of which included the wild-type first AUG codon and a mutated second codon, although the second construct contained the wild-type second AUG codon and a mutated first codon. Both yielded functionally recombinant proteins, leading to the conclusion that *B. burgdorferi* Bpur is naturally translated from the two start codons. EMSA studies described below indicated that both sizes of Bpur bound nucleic acids with indistinguishable affinities. The smaller form, using the second initiation codon, was used for the following studies of Bpur function.

**FIGURE 4.**
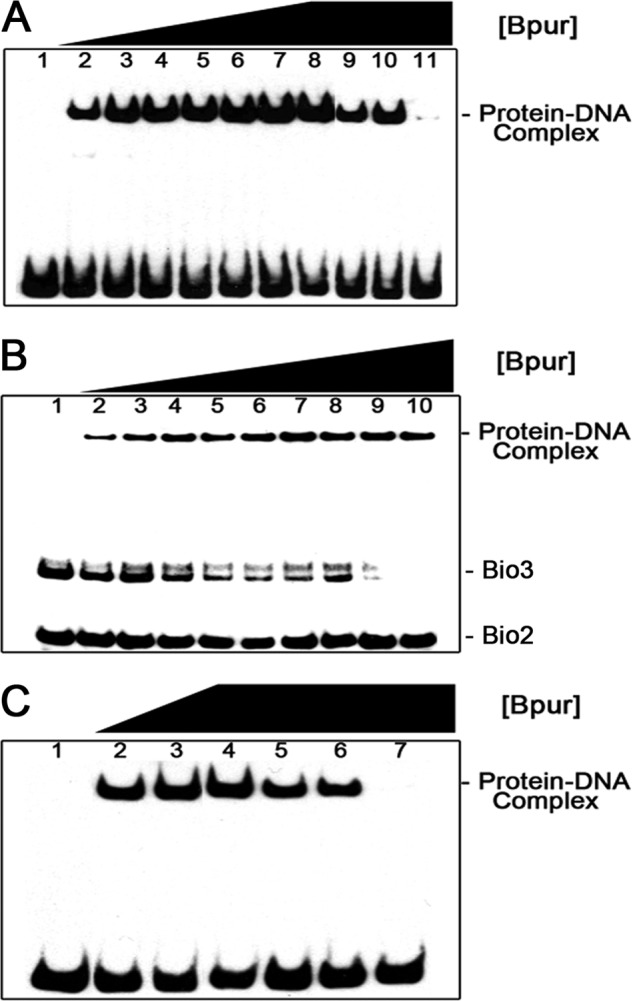
*A,* EMSA of interactions between recombinant Bpur and *erp* 5′-noncoding dsDNA. All lanes contained 2 nm biotin-labeled DNA probe Bio-1. *Lane 1,* no added protein. *Lanes 2–8* contained increasing amounts of Bpur, increasing stepwise from 50 to 500 nm, indicating dose-dependent binding by Bpur. *Lane 9,* 500 nm BpuR plus 100-fold molar excess competitor A. *Lane 10,* 500 nm Bpur plus 100-fold molar excess competitor B. *Lane 11,* 500 nm Bpur plus 100-fold molar excess competitor C. Those data indicate that competitors A and B each contain a portion of the Bpur-binding sequence, although competitor C, which spans the junction of competitors A and B, includes the full Bpur-binding sequence (Fig. 2). *B,* further definition of the Bpur-binding site by simultaneous EMSA using 1 nm each of labeled probes Bio-2 and Bio-3. Probe Bio-3 contains all the sequence of Bio-2, plus an additional 19 bp (Fig. 2). *Lane 1,* DNAs alone. *Lanes 2–10,* increasing concentrations of Bpur. The protein preferentially bound probe Bio-3, indicating that the Bpur-binding site of the *erp* operator is contained within the unique sequence of that probe. *C,* additional competition EMSAs to further refine the Bpur-binding site. All lanes contain 2 nm labeled probe Bio-1. *Lane 1,* no added protein. *Lanes 2–4,* 50, 100, and 250 nm Bpur, respectively. *Lane 5,* 250 nm Bpur and 100-fold molar excess of DNA competitor D. *Lane 6,* 250 nm Bpur and 100-fold molar excess of DNA competitor E. *Lane 7,* 250 nm Bpur and 100-fold molar excess of DNA competitor C.

EMSAs with purified recombinant Bpur and combinations of labeled probes and unlabeled dsDNA competitors were used to confirm that this protein bound *erp* operator DNA and to map the Bpur-binding site (Figs. 1 and 4). Recombinant Bpur bound in a dose-dependent manner to labeled probe Bio-1, which includes the entire *erp* operator region (Fig. 4*A*, *lanes 2–8*). Unlabeled competitor C eliminated Bpur binding to Bio-1, whereas competitors A and B each reduced Bpur binding, indicating that the high affinity binding site is contained within competitor C and partly within competitors A and B (Fig. 4*A*, *lanes 9–11*). Labeled probe Bio-3 includes 22 bp not contained in probe Bio-2 (Fig. 1). Bpur bound to probe Bio-3 but not Bio-2 (Fig. 4*B*). The 22-bp extension of Bio-3 is also contained in competitor C (Fig. 1). Unlabeled competitors D and E are each identical to competitor C except for 7 or 8 bp of the identified 22 bp, respectively (Fig. 1). Competitors D and E partly inhibited Bpur binding to probe Bio-1, whereas competitor C eliminated binding (Fig. 4*C*, *lanes 5–7*). Thus, the 22-bp dsDNA sequence within the *erp* operator was identified as being necessary and sufficient for optimal Bpur binding.

##### Bpur Enhances Activity of the BpaB erp Repressor Protein

**FIGURE 5.**
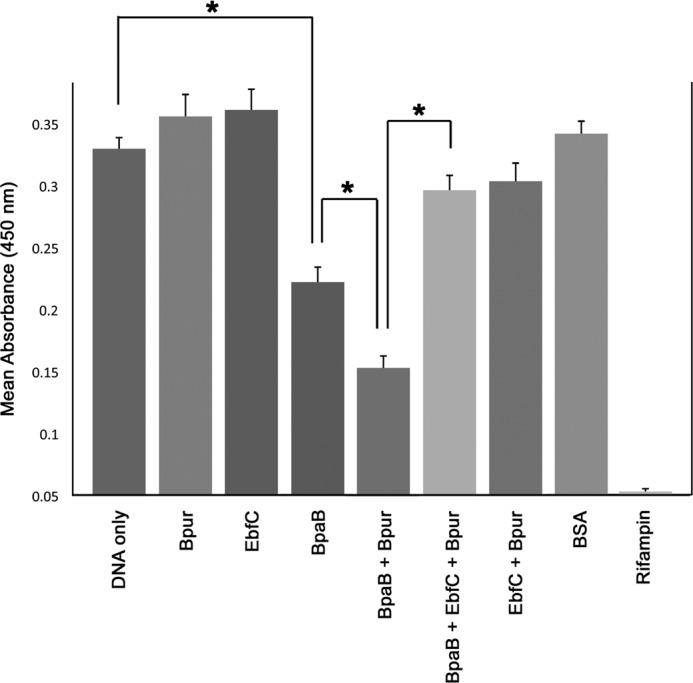
**Effects of purified Bpur, BpaB, and EbfC proteins on *erp* expression in a coupled *in vitro* transcription/translation system.** Product levels were quantified by ELISA and are reported as mean absorbance of three independent experiments. *Asterisks* indicate statistically significant differences (*p* < 0.001 by Student's *t* test) between DNA only and BpaB added, between BpaB alone and BpaB plus Bpur, and between BpaB plus Bpur and inclusions of all three proteins. BSA served as a control to confirm that results were specific for each protein. Addition of rifampin completely prevented product formation, demonstrating that results were dependent upon transcription.

*In vitro* transcription-translation assays were utilized to determine the effect of Bpur on *erp* expression (Fig. 5) (20). Use of an operon fusion between the *erpAB* promoter/regulatory DNA and *gfp* permits efficient analyses of transcriptional activity through quantification of the reporter green fluorescent protein (20). Addition of Bpur alone did not significantly alter promoter activity, indicating that the effects of Bpur and BpaB combined were mediated through the BpaB repressor as described below. The lack of effect by Bpur alone also indicated that it did not impact translation of *gfp* due to Bpur's RNA binding activity (as described below). As reported previously, addition of BpaB inhibited transcription (20). The combination of equimolar Bpur and BpaB significantly inhibited transcription even further. We previously reported that equimolar concentrations of BpaB and EbfC yield transcript levels essentially the same as if neither protein was included, indicating that EbfC in an antirepressor (20). Similarly, equimolar concentrations of Bpur, BpaB, and EbfC yielded expression levels that were not significantly different from experiments that lacked all three DNA-binding proteins (Fig. 5). Inclusion of EbfC and Bpur together did not significantly change expression levels. Control studies with BSA demonstrated that none of these effects were due merely to addition of extraneous protein into the *in vitro* transcription-translation reactions, and addition of rifampin showed that transcription was necessary for assay readout. These data indicate that Bpur enhances the repressive effect of BpaB and that EbfC is able to counteract the combined effects of Bpur and BpaB.

**FIGURE 6.**
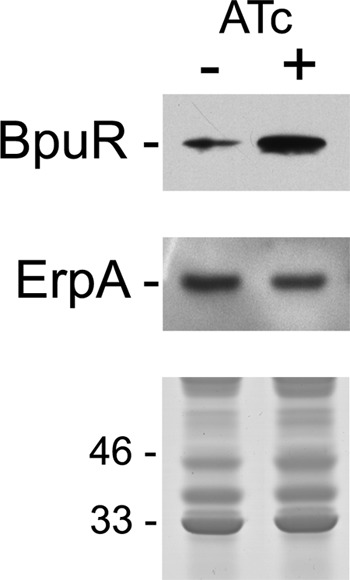
**Effects of increased concentrations of Bpur in *B. burgdorferi*, using an anhydrotetracycline (*AT*)-inducible promoter system.** Levels of Bpur and ErpA in uninduced (−) and induced (+) bacteria were determined by immunoblot. The *lower panel* illustrates SDS-PAGE of each bacterial lysate, stained with Coomassie Brilliant Blue, to confirm equal loading in each lane. Positions of molecular mass standards are shown to the *left* of the stained gel.

Repeated attempts to delete the *bpur* gene, using standard allelic exchange methods (75, 76), have not been successful, suggesting that Bpur may be essential for *B. burgdorferi* survival. As an alternative approach to examine the *in vivo* effects of specifically altered cellular concentrations of Bpur, an inducible promoter system was instead used to enhance production of that protein (19, 20, 62). Induction of Bpur production was accompanied by significant reductions in bacterial Erp protein levels (Fig. 6).

**FIGURE 7.**
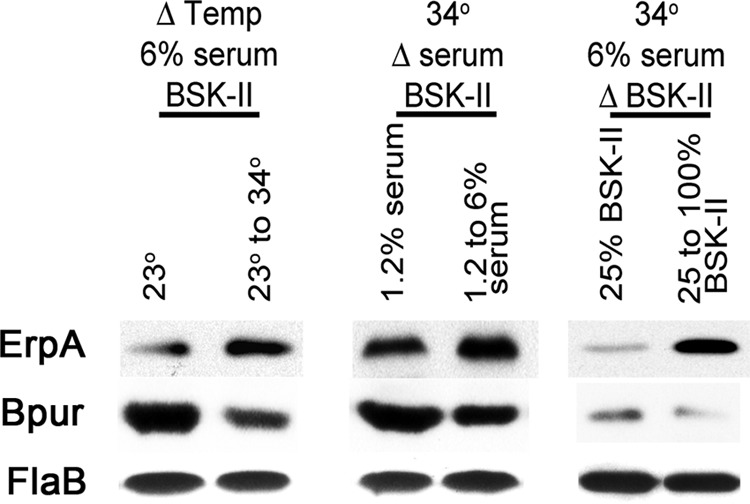
**Changes in *B. burgdorferi* growth rate correlate with changed cellular concentrations of Bpur and Erp proteins.** Immunoblot analyses of the effects of changing *B. burgdorferi* culture conditions on protein expression levels are shown. For each column, two conditions were kept constant, although a third condition was altered. For all studies, bacteria were first cultured to late exponential phase under a condition that impaired growth (complete BSK-II medium at 23 °C, BSK-II containing only 1.2% rabbit serum at 34 °C, or 25% strength BSK-II with 6% serum at 34 °C), then diluted 1:100 into fresh and complete BSK-II, and cultured at 34 °C. All cultures were harvested at late exponential phase. The constitutively expressed flagellar component FlaB served as a control. Illustrated data for each condition are from analyses of the same paired bacterial lysates.

Rapidly dividing bacteria produce greater levels of Erp proteins than do slowly growing bacteria (66). Differences in growth rate can be achieved in culture by changing the incubation temperature or by culture in either incomplete or complete media while maintaining a constant temperature (65, 66, 77). Previous studies found that culture conditions that result in high levels of Erp production are accompanied by high levels of the antirepressor EbfC and low levels of the repressor BpaB, although conditions that reduce Erp levels correlate with low levels of EbfC and high levels of BpaB (66). Repeats of those culture studies determined that cellular concentrations of Bpur were inversely correlated with multiplication rate, with higher levels of Bpur being produced during conditions of slow bacterial growth and division (Fig. 7). Thus, the above-described *in vivo* and *in vitro* studies of Bpur are all consistent with that protein being a co-inhibitor of *erp* expression, acting in conjunction with the repressor BpaB.

##### Characteristics of Bpur-Nucleic Acid Interactions

The mechanisms by which PUR domains interact with nucleic acids are poorly characterized. Therefore, protein-ligand studies were undertaken with Bpur to provide the groundwork necessary for developing models of that protein's impact on *erp* transcription. In addition, these data yielded new insight on PUR domains in general.

**FIGURE 8.**
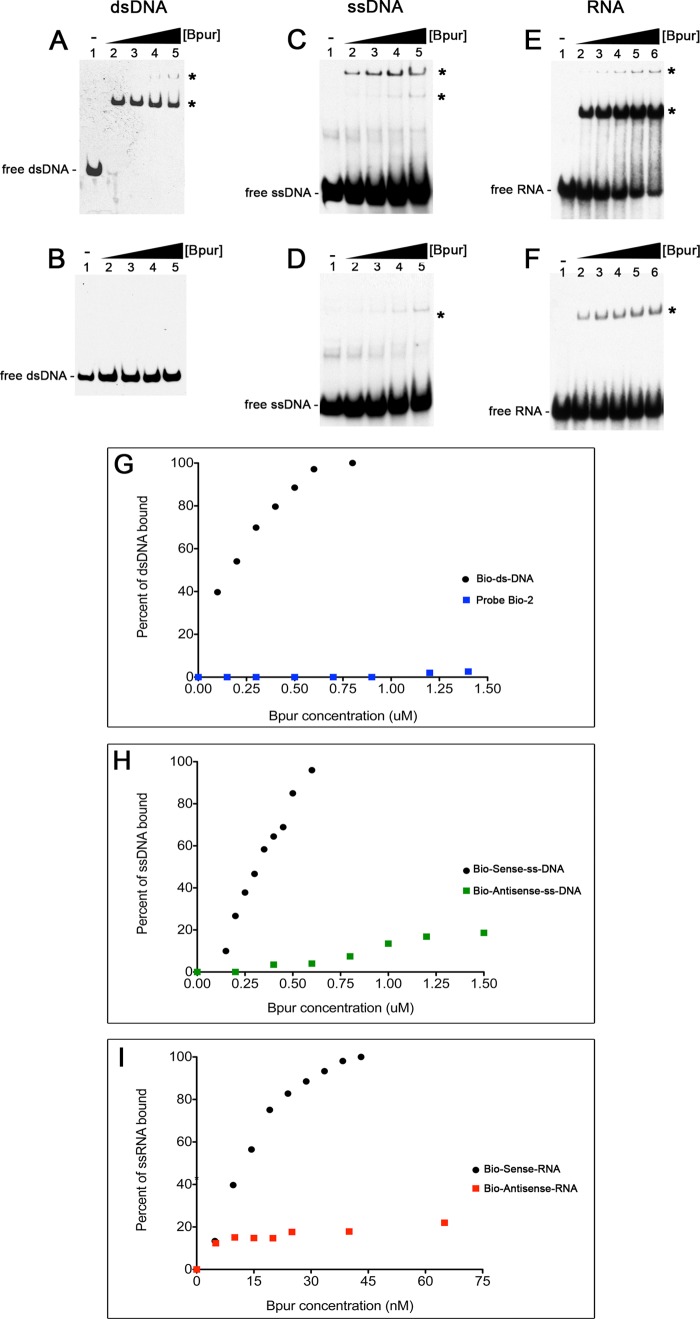
**Site specificity and nucleic acid preferences of Bpur.** *A–F* show EMSAs of wild-type recombinant Bpur and various labeled nucleic acids (Table 1). The nucleic acid probe sequences used in *A* and *C–F* were 54 bp/bases in length and were based on the high affinity Bpur-binding site in the *B. burgdorferi erp* operator. Bpur exhibited different affinities for each ligand, so the EMSAs in each panel contained different ligand and protein concentrations to illustrate binding or lack thereof. *A,* 2.5 nm labeled probe Bio-dsDNA incubated with 0, 100, 200, 300, or 500 nm Bpur, *lanes 1–5*, respectively. *B,* control, consisting of an unrelated 54-bp dsDNA-labeled probe that was based on the sequence of the *B. burgdorferi erpA* open reading frame and incubated with 0, 100, 200, 300, or 500 nm Bpur, *lanes 1–5*, respectively. *C,* 7.5 nm labeled probe Bio-Sense-ssDNA, incubated with 0, 100, 200, 300, or 500 nm Bpur, *lanes 1–5*, respectively. *D,* 7.5 nm labeled probe Bio-antisense-ssDNA, incubated with 0, 100, 200, 300, or 500 nm Bpur, *lanes 1–5*, respectively. *E,* 2 nm labeled probe Bio-sense-RNA, incubated with 0, 2, 4, 8, or 12 nm Bpur, *lanes 1–5*, respectively. *F,* 2 nm labeled probe Bio-antisense-RNA, incubated with 0, 2, 4, 8, or 12 nm Bpur, *lanes 1–5*, respectively. *G–I,* relative intensities of Bpur-nucleic acid complexes graphed for dsDNA, ssDNA, and RNA, respectively.

**FIGURE 9.**
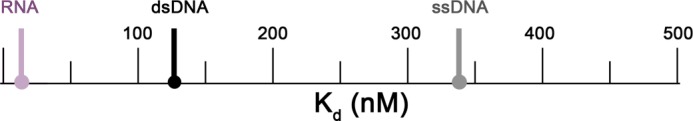
**Graphic representation of the dissociation constant (*K_D_*) values of Bpur interactions with each nucleic acid ligand: RNA *K_D_* = 13 nm, dsDNA *K_D_* = 130 nm, and ssDNA *K_D_* = 390 nm.** *K_D_* values were determined by analyses of EMSA gel images, with ratios of bound/free DNA in each lane, and calculated as described previously (18, 58–60).

The ends of linearized dsDNA are physically different from internal regions, and these end effects extend inward for one helical turn, or ∼10 bp (78). For that reason, these detailed analyses of interactions between Bpur and nucleic acids utilized 54-base/base pair ligands based on the *erp* operator, with a centrally located binding site (Fig. 1 and Table 1). Recombinant Bpur bound to a 54-bp dsDNA fragment of the *erp* operator but not to other tested dsDNAs (Figs. 4 and 8, *A, B,* and *G*). Comprehensive binding analyses determined that Bpur bound the *erp* operator probe with a *K_D_* = 130 nm (Fig. 9).

**TABLE 1:** **Sequences of nucleic acid probes used to determine the relative affinities of Bpur for dsDNA, ssDNA, and RNA** Only one strand of the dsDNA is listed. The sequences were based on the identified Bpur-binding site with the *erp* operator. The 22-base sequence identified as necessary and sufficient for Bpur-binding is underlined.

Name	Sequence (5′→3′)
Bio-dsDNA (dsDNA)	AATGGAGAGATTTTGGGGAGTTGTTTAAAATTACATTTGCGTTTTGTTAAAATG
Bio-sense-ssDNA	AATGGAGAGATTTTGGGGAGTTGTTTAAAATTACATTTGCGTTTTGTTAAAATG
Bio-antisense-ssDNA	CATTTTAACAAAACGCAAATGTAATTTTAAACAACTCCCCAAAATCTCTCCATT
Bio-sense-RNA	AAUGGAGAGAUUUUGGGGAGUUGUUUAAAAUUACAUUUGCGUUUUGUUAAAAUG
Bio-antisense-RNA	CAUUUUAACAAAACGCAAAUGUAAUUUUAAACAACUCCCCAAAAUCUCUCCAUU

Because eukaryotic PUR domain proteins also bind ssDNA and RNA, the ability of Bpur to interact with those types of nucleic acids was examined. The 54-base high affinity sequence described above was again used for these analyses, with each nucleic acid strand being assayed separately. Bpur exhibited a greater affinity for the purine-rich ssDNA probe than it did for the complementary pyrimidine-rich probe (Fig. 8, *C*, *D*, and *H*). Probe Bio-Sense-ssDNA was bound with a *K_D_* = 390 nm, whereas the affinity for the complementary sequence was significantly weaker, and a *K_D_* value could not be determined (Fig. 9). Likewise, Bpur bound to the purine-rich Bio-Sense-RNA probe but exhibited a markedly lower affinity for the complementary pyrimidine-rich sequence (Fig. 8, *E, F,* and *I*). Bpur exhibited the highest affinity of all for the purine-rich RNA probe, with a calculated *K_D_* = 13 nm (Fig. 9). In addition to defining sequence specificity of Bpur, these studies were the first analyses to determine the relative affinities of any PUR domain for all types of ligands.

**FIGURE 10.**
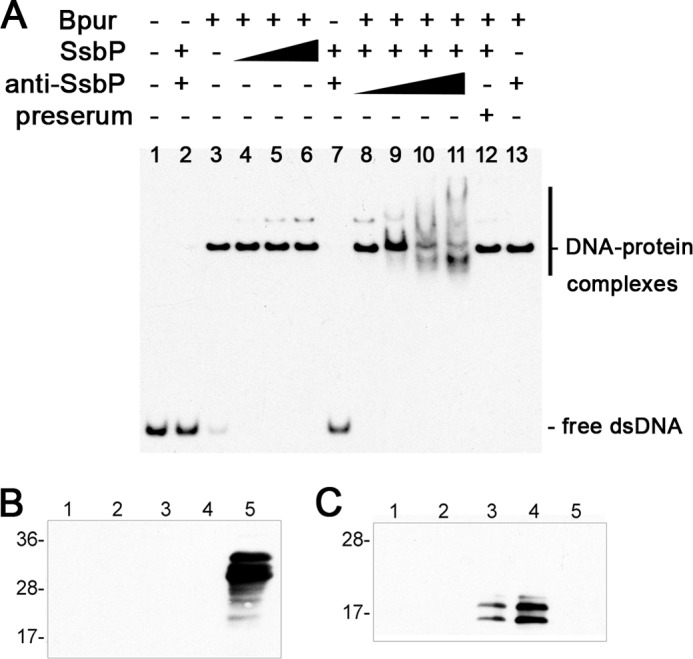
**Bpur separates the strands of dsDNA.** *A,* EMSA data of Bpur interacting with labeled 54-bp dsDNA probe Bio-1. SsbP specifically binds only ssDNA, without nucleotide sequence preference (19). *Lane 1,* 2.5 nm DNA alone. *Lane 2,* DNA + SsbP ssDNA-binding protein. *Lane 3,* DNA + 200 nm Bpur. *Lanes 4–6,* DNA + 200 nm Bpur + SsbP at concentrations of 1, 2, or 5 nm, respectively. *Lane 7,* DNA + SsbP + SsbP-specific antiserum. *Lanes 8–11,* DNA + 200 nm Bpur + 5 nm SsbP + increasing concentrations of SsbP-specific antiserum. *Lane 12,* DNA + SsbP + preserum. *Lane 13,* DNA + Bpur + SsbP-specific antiserum. By itself, SsbP did not bind dsDNA (*lanes 2* and *7*), as reported previously (19). Addition of SsbP to Bpur-complexed dsDNA yielded a second, dose-dependent protein-DNA complex (*lanes 4–6*). Confirmation that this complex included SsbP was provided by co-incubation with SsbP-specific antibodies (anti-SsbP), which produced a supershift (*lanes 8–11*). Control studies with preimmune serum (preserum) or antiserum alone confirmed the specificity of these results (*lanes 12* and *13*, respectively). *B* and *C,* immunoprecipitation analyses demonstrating that Bpur and SsbP proteins do not interact with each other. All five lanes of each panel contained aliquots of the same preparations. *Lanes 1*, mock immunoprecipitation reaction with irrelevant IgG-conjugated beads; *lanes 2,* mock immunoprecipitation using plain beads; *lanes 3,* immunoprecipitation with anti-Bpur antibodies conjugated to beads; *lanes 4,* purified rBpur protein; *lanes 5,* purified SsbP protein. *B,* immunoblot probed with SsbP-specific antibodies. *C,* immunoblot probed with Bpur-specific antibodies.

Bpur was further demonstrated to separate the strands of dsDNA, a feature shared with human Pur-α (Fig. 10) (79). To facilitate these studies, we employed the *B. burgdorferi* SsbP protein (single-stranded DNA-binding protein of the prophage). This protein binds ssDNA with high affinity and low specificity, but it does not detectably bind dsDNA (Fig. 10*A*, *lane 2*) (19). Incubation of SsbP with Bpur-complexed dsDNA produced an additional protein-DNA complex (Fig. 10*A*, *lanes 3–6*). Inclusion of SsbP-specific antibody yielded an EMSA supershift, demonstrating that the second complex contained SsbP (Fig. 10*A*, *lanes 8–11*). Control studies demonstrated that SsbP and Bpur do not directly interact with each other (Fig. 10, *B* and *C*), indicating that the additional EMSA complexes in Fig. 10*A* were due to SsbP-ssDNA interactions.

##### Identification of Bpur Residues Involved with Nucleic Acid Binding

The Bpur PUR domain was subjected to molecular analyses, to further understand how it interacts with nucleic acids and could thereby affect borrelial physiology. Because little is known about the mechanisms by which any PUR domain protein binds nucleic acids, these results also shed light on all proteins that contain this motif. Based upon the solved structure of Bpur (22), rational site-directed mutagenesis was used to produce a series of mutant proteins. Specific amino acid residues were mutated that met the following criteria: 1) structurally conserved between *B. burgdorferi* Bpur and eukaryotic PUR domains; 2) not directly involved with Bpur secondary or tertiary structure, and 3) exposed to the environment and thereby available to interact with ligands. In addition, amino-terminal truncations were produced, because a previous study indicated involvement of the human Pur-α amino terminus in binding to ssDNA (23). All specifically mutated Bpur residues are described in Table 2, and locations of mutations that affected Bpur functions are depicted in Fig. 2. Of these, three mutations had significant effects on the binding of one or more, but not all, substrates. Those differences indicate distinct interactions with each type of ligand. Because each of these mutations did not disturb interactions with at least one ligand, it is clear that the effects were not simply due to gross protein misfolding.

**TABLE 2:** **Recombinant Bpur proteins used in these studies**

Recombinant protein	Description	Effect on nucleic acid binding?
Bpur N+5	Full length wild-type protein initiating from first AUG codon	No effects (data not shown)
Bpur	Full length wild-type protein initiating from second AUG codon	No effects
Bpur ΔN-12	Deletion of the first 12 amino acids from the amino terminus	No effects (data not shown)
Bpur ΔN	Deletion of the first 28 amino acids from the amino terminus	Yes
Bpur ΔC	Deletion of the last 36 amino acids from the carboxyl terminus	No effects (data not shown)
Bpur E63A/S64A	Amino acid substitutions E63A/S64A	Yes*^a^*
Bpur L67A	Amino acid substitutions L67A	No effects (data not shown)
Bpur L68A/K69A	Amino acid substitutions L68A/K69A	No effects (data not shown)
Bpur Q75A	Amino acid substitutions Q75A	Yes*^a^*

*^a^* See text for details of the impacts of these mutations on ligand binding.

**FIGURE 11.**
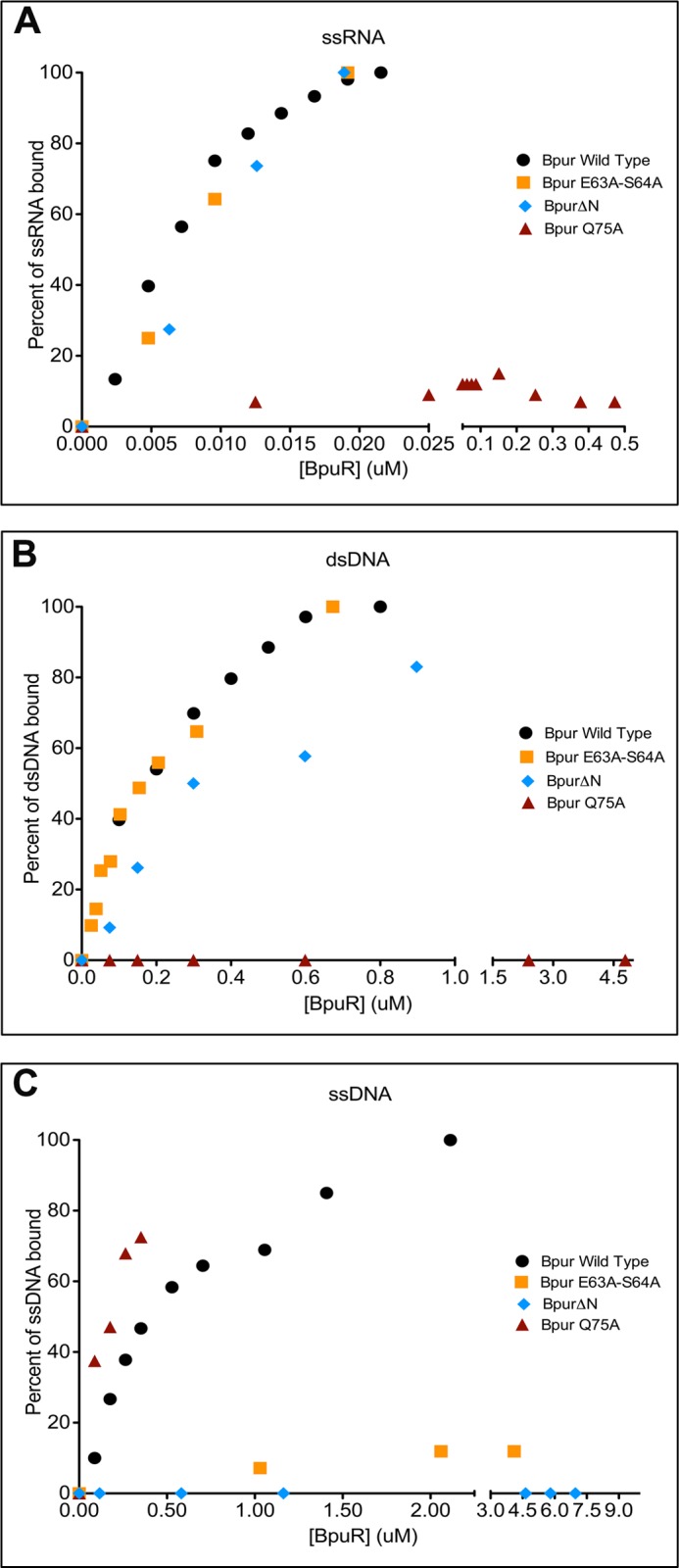
**Different regions of the PUR domain are involved with binding each type of nucleic acid.** *A–C* illustrate representative nucleic acid-binding experiments of wild-type and specifically mutated Bpur proteins binding to RNA, dsDNA, and ssDNA, respectively. Binding affinity analyses (18) determined the following *K_D_* values for each protein: RNA, wild-type Bpur = 13 nm, Bpur-Δ*n* = 12 nm, Bpur-E63A/S64A = 15 nm, and Bpur-Q75A > 650 nm; dsDNA, wild-type Bpur = 127 nm, Bpur-Δ*n* = 268 nm, Bpur-E63A/S64A = 137 nm, and Bpur-Q75A > 6 μm; ssDNA, wild-type Bpur = 393 nm, Bpur-ΔN > 6 μm, Bpur-E63A/S64A > 10 μm, and Bpur-Q75A = 279 nm.

Mutant Bpur-Q75A contains a substitution of glutamine 75 to alanine, near the α-helix's carboxyl end. Bpur-Q75A exhibited significantly reduced affinity for both dsDNA and RNA, with negligible binding observed even when using the highest possible concentrations of protein, and *K_D_* values could not be calculated for either ligand (Fig. 11, *A* and *B*). In contrast, the affinity of Bpur-Q75A for ssDNA was not significantly different from that of the wild-type protein (Fig. 11*C*).

Opposite effects were observed with mutant Bpur-E63A/S64A, which contains alanines substituted for glutamate 63 and serine 64, near the amino end of the α-helix. Bpur-E63A/S64A bound both dsDNA and RNA with the same affinities as did the wild-type protein, yet did not detectably bind ssDNA (Fig. 11, *A–C*).

Even more striking was mutant Bpur-ΔN, which lacks the two amino-terminal β-strands. This mutation removed 23% of the Bpur protein, yet did not have any significant effects upon binding to RNA, and it reduced the affinity for dsDNA by only 2-fold (Fig. 11, *A* and *B*). However, Bpur-ΔN did not detectably bind ssDNA (Fig. 11*C*).

**FIGURE 12.**
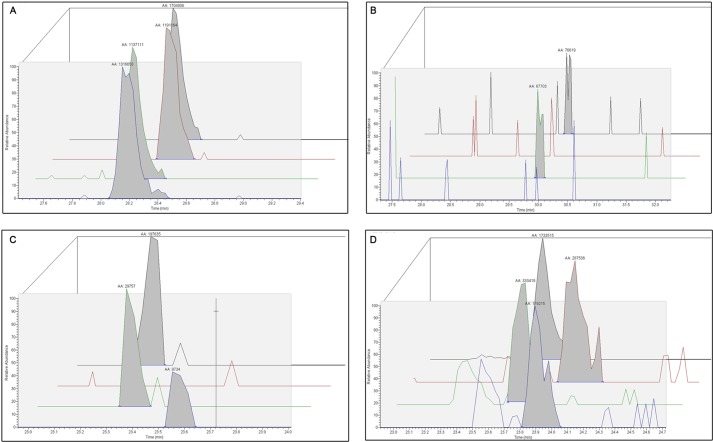
**Illustrative examples of quantitative MS/MS data mined from limited proteolysis assay.** All tryptic polypeptides, identified by a MASCOT search via Proteome Discoverer 1.3, were mapped against the linear amino acid sequence of Bpur for each time point and substrate group. Ion scores pertaining to individual polypeptide concentrations and the confidence of polypeptide identification through LTQ Velos Orbitrap mass coupled with a nano-LC Ultra/cHiPLC-nanoflex HPLC system are shown. Extracted ion chromatograms for polypeptides surrounding a given cleavage site were evaluated for signal intensity and peak area. Relative peak intensity (arbitrary units) is measured on the *y* axis, and peptide retention time (minutes) is measured on the *x* axis. Four representative data sets are presented. *Black lines,* no substrate added. *Red lines,* Bpur bound to dsDNA. *Green lines,* Bpur bound to ssDNA. *Purple lines,* Bpur bound to RNA. *A,* representative data set showing results when binding of a ligand did not change a rate of digestion, relative to the Bpur alone control reactions: TYFFNVK polypeptide presence after 20 min of incubation with trypsin. *B,* levels of the GDYFLNIVESK polypeptide after 10 min of incubation with trypsin. Binding of RNA or dsDNA inhibited cleavage adjacent to residue Lys-40. *C,* levels of the QKVSTGSVGSSAR polypeptide after 20 min of incubation with trypsin. To different extents, binding of each ligand inhibited cleavage at residue Lys-74. *D,* levels of the RSPSGDFERAIAVIK polypeptide after 20 min of incubation with trypsin. To different extents, each nucleic acid ligand decreased cleavage adjacent to residue Lys-40, resulting in the increased production of a larger polypeptide fragment.

**FIGURE 13.**
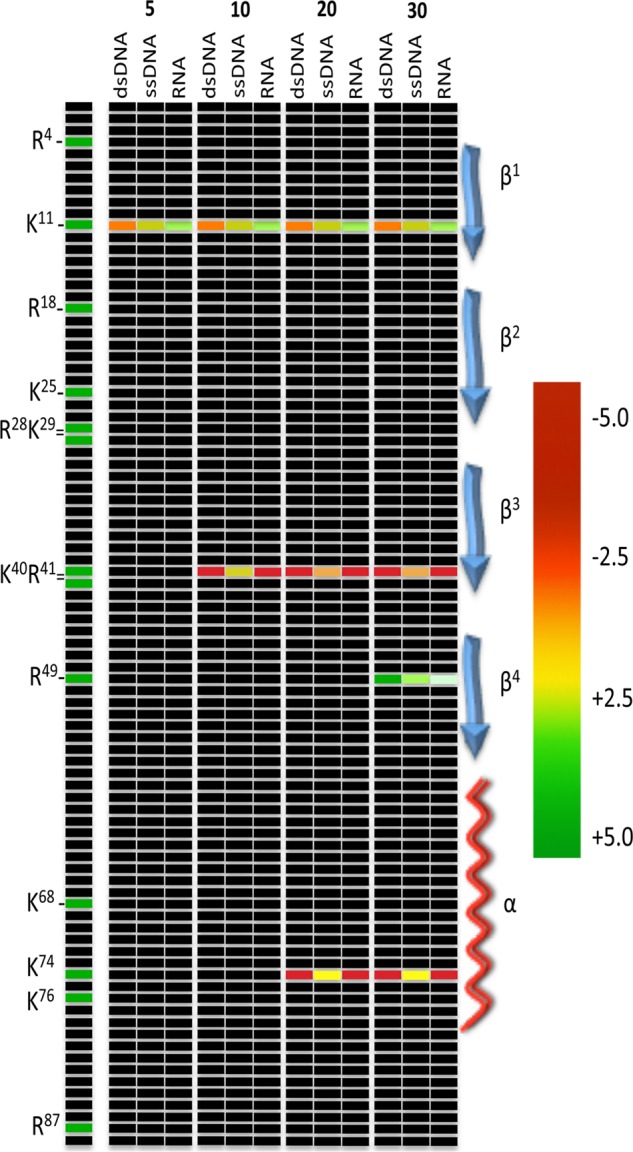
**Graphic representation of changes in protease accessibility to Bpur sites that were induced by binding of a nucleic acid.** Recombinant Bpur was prebound with saturating concentrations of dsDNA, ssDNA, or RNA and then subjected to proteolysis by trypsin for 5, 10, 20, or 30 min. Reaction products were analyzed by quantitative LC-MS/MS, and relative polypeptide concentrations were compared based on signal intensities and areas under the peaks. Each cleavage site and digestion incubation time was assayed as a function of fold change. Normalized values were log_2_-transformed. Frequencies of cutting adjacent to each amino acid of the PUR domain was diagrammed to illustrate the three ligands' relative effects on trypsin cutting. *Red* indicates impaired protease cleavage and *green* indicates enhanced cutting. All trypsin-cleavage sites of the PUR domain are listed to the *left* of the panel. Sites unaffected by a bound ligand are marked in *black*. Locations of Bpur protein secondary structures are diagrammed to the *right*.

Binding of a ligand by a protein can alter *in vitro* access of protease to potential cleavage sites by obscuring or exposing sites. Limited-duration proteolysis can thus reveal regions of a protein that either interacts directly with a ligand or changes conformation upon an induced ligand fit (80–82). Effects of ligand binding on Bpur were assessed by first saturating the free protein with either RNA, ssDNA, or dsDNA and then incubating with trypsin for short intervals, followed by polypeptide fragment composition quantification using Orbitrap mass spectrometry. The use of Orbitrap technology permitted very high levels of sensitivity and quantification (83). By comparison and normalization against results obtained from Bpur alone, relative levels at which trypsin cleaved each potential site were calculated. Examples of data are illustrated in Fig. 12. Nine of the 13 trypsin cleavage sites in the PUR domain were unaffected by any ligand, thereby serving as additional internal controls. Significant changes in protease cleavage at the other four trypsin sites of Bpur were observed (Fig. 13).

All three ligand types protected cleavage adjacent to lysine 74 of the α-helix, although the levels of protection afforded by dsDNA or RNA were markedly greater than that of ssDNA (Fig. 13). This trypsin cleavage site is adjacent to the glutamine 75 residue that is essential for binding dsDNA and RNA, but not ssDNA, further supporting a role for the carboxyl end of the α-helix in binding those ligands.

Cleavage adjacent to arginine 49 was enhanced when Bpur bound ds- or ssDNA (Fig. 13). Those results are consistent with the previously reported involvement of that residue with binding to ssDNA (23). In contrast, binding of RNA only slightly enhanced digestion adjacent to arginine 49. The trypsin site adjacent to the nearby lysine 40 was strongly protected by both dsDNA and RNA and to a lesser extent by ssDNA. Cleavage adjacent to lysine 11 was enhanced by RNA and ssDNA but impaired by dsDNA. Thus, limited proteolysis/mass spectrometry analyses indicated that ligand binding induced structural changes in the β-stranded region of the PUR domain and that such changes were distinct for each nucleic acid ligand.

## DISCUSSION

The complex life cycle of *B. burgdorferi* requires that the spirochete produce many different host-interactive surface proteins at distinct, precise locations. It therefore came as a surprise when sequencing of the *B. burgdorferi* genome revealed an apparent paucity of regulatory factors (55). Since that time, however, several previously undescribed types of regulatory factors have been characterized, such as EbfC and BpaB. That list can now be expanded to include Bpur. Noting that Bpur is encoded on the *B. burgdorferi* main chromosome, whereas *erp* operons are carried on bacteriophages, it is highly likely that Bpur serves additional functions for the Lyme disease spirochete. The affinity of the borrelial PUR domain protein for both DNA and RNA suggests that it can affect both transcription and translation.

Determining the biophysical properties of interactions between Bpur and nucleic acids were essential steps toward defining the precise mechanism(s) through which Bpur enhances repression of *erp* transcription. For example, the propensity of Bpur to open up dsDNA raises possibilities that the protein alters local supercoiling and/or other conformational aspects. Those and other hypotheses are currently being tested to explain the combined effects of Bpur, BpaB, and EbfC on *erp* expression.

It is also notable that a wide range of other Eubacterial species produce two of the nucleic acid-binding proteins identified from studies of the *B. burgdorferi erp* operons, Bpur and EbfC. Thus, studies of these borrelial proteins have broad implications for understanding general bacterial physiology, as well as identifying targets for development of novel antibacterial therapies.

These studies provided data consistent with earlier studies of eukaryotic PUR domain proteins, while also greatly increasing the understanding of mechanisms by which a PUR domain interacts with different nucleic acids. New details can now be added to models describing those protein-ligand interactions.

These studies demonstrated that the PUR domain α-helix is critically involved in ligand binding, although different regions of that structure are essential for different ligands. The Q75A and E63A/S64A mutations are each predicted to strengthen the α-helix (84). Additionally, those mutations replaced highly interactive amino acids with alanine, which generally interacts poorly with nucleic acids (85). Notably, both ends of the α-helix in eukaryotic and prokaryotic PUR domains contain solvent-exposed amino acid residues that favorably act as both hydrogen donors and acceptors (22, 23, 85). Collectively, the data suggest that flexibility of the α-helix is important for ligand binding and/or that the ends of the α-helices form intimate contacts with nucleic acids.

All types of nucleic acid ligands induced conformational changes in the β-strand region of Bpur. In addition, binding of ssDNA required α-helix residues 63–64, but not residue 75, plus the amino-terminal β-strands. Glutamine 75 was essential for binding dsDNA but not ssDNA, suggesting that the carboxyl end of the α-helix binds one or both strands. RNA binding did not require either the amino-terminal β-strands or residues 63–64 but did require the carboxyl-end of the α-helix, suggesting that RNA is bound through a mechanism similar to that of dsDNA.

In summary, Bpur was purified as a consequence of its high affinity for *erp* operator dsDNA and was determined to enhance the effects of the BpaB repressor on *erp* transcription. Further investigations revealed that Bpur also binds ssDNA and RNA, with a marked preference for RNA. In addition, this small homodimeric PUR domain protein was shown to serve as a model to allow detailed investigations that cannot be efficiently performed with larger, more complex eukaryotic homologs. These studies revealed important insight into the mechanisms by which a PUR domain interacts with nucleic acids, identifying protein microdomains that interact with each substrate type. The PUR domain is a key component of regulatory proteins that are widely spread across nature. Studies of unicellular model organisms and simple proteins, such as *B. burgdorferi* and Bpur, can provide valuable insight on molecular mechanisms used throughout all domains of life.

## Footnotes

2 The abbreviations used are:

Erp: outer surface protein EF-related protein
Bpur: borrelial PUR domain protein
EbfC: *erp*-binding factor, chromosomal
BpaB: borrelial ParB analog
dsDNA: double-stranded DNA
ssDNA: single-stranded DNA
SsbP: single-stranded DNA-binding protein of the cp32 prophage
anti-SsbP: SsbP-specific antiserum.
